# Nuclear morphological characterisation of lobular carcinoma variants: a morphometric study

**DOI:** 10.1111/his.15390

**Published:** 2024-12-09

**Authors:** Ayaka Katayama, Shorouk Makhlouf, Michael S Toss, Tetsunari Oyama, Emad A Rakha

**Affiliations:** ^1^ Diagnostic Pathology Gunma University Graduate School of Medicine Maebashi Japan; ^2^ Academic Unit for Translational Medical Sciences, School of Medicine University of Nottingham Nottingham UK; ^3^ Department of Pathology, Faculty of Medicine Assiut University Assiut Egypt; ^4^ Department of Histopathology Sheffield Teaching Hospitals, NHS Foundation Trust Sheffield UK; ^5^ Department of Histopathology Nottingham University Hospitals Nottingham UK; ^6^ Department of Pathology Hamad Medical Corporation Doha Qatar

**Keywords:** breast, breast cancer, lobular carcinoma, nuclear size

## Abstract

**Background and aims:**

Lobular carcinoma (LC) of the breast exhibits diverse morphology and clinical behaviour. The pleomorphic variant (pLC) displays distinct cytonuclear features and aggressiveness compared to the classic variant (cLC). However, diagnosing pLC remains subjective. This study aims to refine LC's cytonuclear features, focusing on pLC.

**Methods:**

Whole slide images of 59 LCs, including both *in situ* (LCIS) and invasive (ILC) lesions, were analysed. Nuclear measurements, including nuclear size and variability, were scored using QuPath image analysis software. For comparison, selected features were scored in normal cells (*n* = 10) and pleomorphism score‐matched invasive breast carcinoma (IBC) of NST type (*n* = 33). Additional visual assessment of the pleomorphic ILC (pILC) cohort (*n* = 90) was conducted for cytomorphological features characterisation.

**Results:**

pILC demonstrated larger nuclear area and higher nuclear variability with abundance of cytoplasm than cILC. Compared to lymphocytes, pILC demonstrated a median area ranging from 2.7 to 4.7 times larger. Cut‐off values for differentiating pILC from other ILC subtypes included median nuclear area > 48.2 μm^2^ and interquartile range (IQR) > 19.4, nuclear perimeter median > 25.2 μm and IQR > 5.3 and maximum diameter > 9.1 μm and IQR > 2.2. Multivariable logistic regression confirmed these parameters as independent predictors of pILC, with the maximum diameter being the most significant (*P* < 0.001). Visual assessment recognised two pILC subtypes: apocrine and non‐apocrine. Apocrine variant showed nuclear roundness, pale vesicular chromatin patterns and prominent nucleoli, while non‐apocrine variant exhibited greater nuclear size and shape variation.

**Conclusions:**

Objective nuclear measurements, combined with cytoplasmic and architectural features, provide a robust framework for diagnosing LC subtypes, improving diagnostic accuracy and reproducibility.

## Graphical abstract

This study provides new insights into the diagnosis of lobular carcinoma variants, particularly pleomorphic variants, by refining their cytonuclear features.

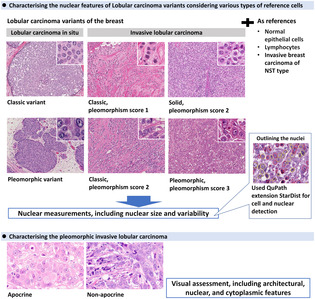

**Abbreviations**
AUC: Area under the curve
cLN: Classic Lobular Carcinoma
DCIS: Ductal carcinoma in situ
H&E: Haematoxylin and eosin
IBC‐NST: Invasive breast carcinoma of no special type
ILC: Invasive Lobular Carcinoma
IQR: Interquartile range
LCIS: Lobular carcinoma in situ
LN: Lobular Carcinoma
NGS: Nottingham grading system
pILC: Pleomorphic Invasive lobular carcinoma
pLC: Pleomorphic lobular carcinoma
pLCIS: Pleomorphic lobular carcinoma in situ
ROI: Region of interest
sILC: Solid Invasive lobular carcinoma
TDLUs: Terminal duct lobular units
WSI: Whole slide image

## Introduction

Lobular carcinoma (LC), which comprises 10–15% of all breast cancers, is characterised by a dyscohesive growth pattern with loss of E‐cadherin membrane expression and/or function.^1^, ^2^, ^3^ Based on cytological and architectural morphologies, LC has recognised variants with distinct clinical behaviour.^2^ Classic LC variant (cLC), the most common variant, usually features small cellular size, condensed monomorphic nuclei and scant cytoplasm, distributed in single cells, single files and targetoid infiltrations. However, variability exists within classic LC, where two cell types were described to represent the spectrum of cytonuclear change or nuclear grade: the classic aforementioned monomorphic type A cells (pleomorphism score 1), and the larger vesicular type B cells (pleomorphism score 2).^4^

Pleomorphic LC (pLC) is a rare special cytomorphological variant of LC, accounting for fewer than 1% of all breast cancers, characterised by higher nuclear‐grade features (pleomorphism score 3), and is associated with a worse prognosis.^2^, ^5^, ^6^, ^7^, ^8^, ^9^, ^10^ The pleomorphic lobular subtype is recognised in both *in situ* (lobular carcinoma *in situ* (LCIS)) and invasive (invasive lobular carcinoma (ILC)) disease. Pleomorphic LCIS (pLCIS) are currently managed as ductal carcinoma *in situ* (DCIS), while classic LCIS (cLCIS) is considered by some authors as an entity that does not need to be staged as carcinoma *in situ*.^5^, ^10^, ^11^ Unlike classic ILC (cILC), pleomorphic ILC (pILC) exhibits more aggressive behaviour surpassing not only that of cILC, but also that of invasive breast cancer of no special type (IBC‐NST); presents at a more advanced stage; and had frequent mutations in *ERBB2* and PIK3CA pathway alternations and shorter survival.^2^, ^12^

As a result of these clinical implications, the distinction between cLC and pLC should be based on defined criteria to ensure a high degree of reproducibility and concordance among pathologists. However, defining the threshold for what constitutes pLC remains a common challenge. The current diagnostic criteria indicate that the cells of pLC are large and markedly pleomorphic, mimicking pleomorphism score 3 of ductal carcinoma using Nottingham grading system (NGS),^5^, ^6^, ^13^ with more abundant cytoplasm than the classic variant.^7^, ^10^ However, such definitions are subjective and comparison with score 3 ductal carcinoma cells in individual cases is often not possible. The concordance of assessment of pleomorphism scores in ductal carcinomas is already low.^14^ It has also been reported that the nuclear size of pLC is three to four times the size of a lymphocyte with a nuclear diameter determined to be ≥ 18 μm, despite lack of evidence‐based studies.^15^ Some authors have defined pLC as cells that are bigger than four lymphocytes,^5^ while other authors have stated that the nuclear size of pLC is at least or more than four times the size of lymphocyte nuclei.^4^ In addition to such variation in the size comparison to lymphocytes, lymphocytes also vary in size, depending upon fixation and processing. However, these features remain subjective and include a wide range, which makes the diagnosis of LC variants showing variable degrees of nuclear pleomorphism more challenging.^14^

To our knowledge, there have been no reports to accurately characterise the nuclear features of LC variants considering the size of various types of reference cells. Computer‐aided image analysis applied to histopathological images has enabled accurate detection and measurement of various cellular and subcellular features in an applicable objective approach. Therefore, with the aid of image analysis, the current study aimed to summarise nuclear features of LC variants, including both *in situ* and invasive lesions, with a comparison to the nuclear size of normal epithelial cells and lymphocytes in addition to the score‐matched IBC‐NST.

## Materials and methods

### Study cohort

**Figure 1:**
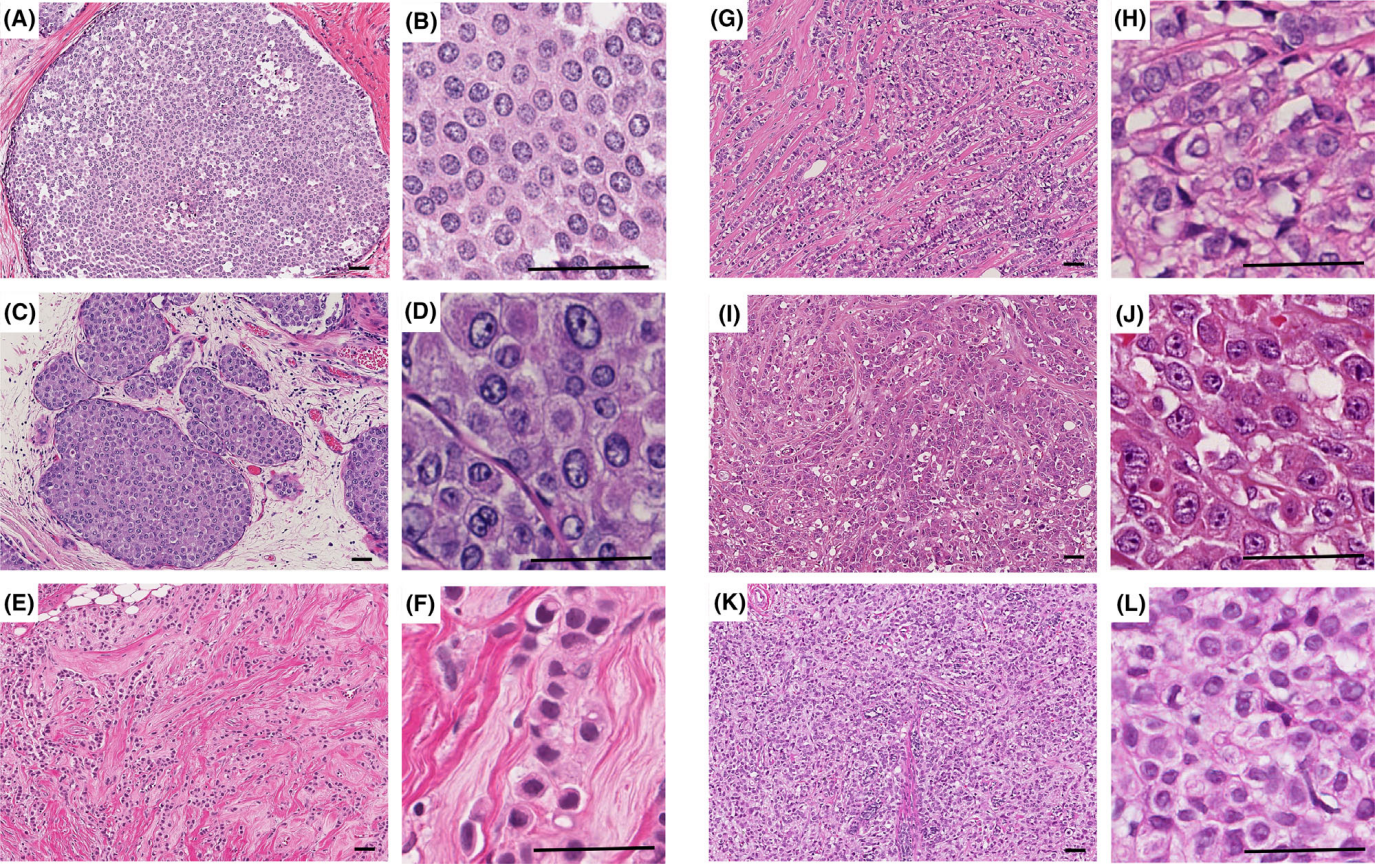
Histological appearances of lobular carcinoma variants included in this study. **A**,**B**, Lobular carcinoma *in situ* (LCIS), classic type. Growth pattern (**A**) and enlarged view of cells (**B**). **C**,**D**, LCIS, pleomorphic type. Growth pattern (**C**) and enlarged view of cells (**D**). **E**,**F**, ILC with classic architecture and pleomorphism score 1. Growth pattern (**E**) and enlarged view of cells (**F**). **G**,**H**, Invasive lobular carcinoma (ILC) with classic architecture and pleomorphism score 2. Growth pattern (**G**) and enlarged view of cells (**H**). **I**,**J**, ILC with classic architecture and pleomorphism score 3, known as pleomorphic variants. Growth pattern (**I**) and enlarged view of cells (**J**). **K**,**L**, ILC with solid architecture and pleomorphism score 2. Growth pattern (**K**) and enlarged view of cells (**L**). Haematoxylin and eosin, scale bar = 50 μm). [Color figure can be viewed at wileyonlinelibrary.com]

Fifty‐nine LCs, including LCIS of classic (*n* = four) and pleomorphic (*n* = four) variants and ILC of classic (*n* = 26), solid (*n* = 11) and pleomorphic (*n* = 14) variants from patients presented at Breast Institute, Nottingham City Hospital, Nottingham, UK were investigated. Their histological features were reviewed and agreed upon by four pathologists (A.K., S.M., M.T. and E.R.) according to the 5th edition of the World Health Organisation (WHO) classification of breast tumours.^5^ Discordant cases were excluded. The selection and subclassification of the various LC lesions were challenging, as some showed a spectrum of changes with overlapping features and some cases showed a mixture of lesions. After histological review, only foci representing each variant were selected for the measurement, while discordant cases and discordant foci were excluded. Pleomorphism score‐matched IBC‐NST were included as a control group (*n* = 33). Nuclear pleomorphism was graded from scores 1 to 3, according to NGS criteria.^16^ Examples of LC variants included in this study are illustrated in Figure 1. Normal epithelial ductal cells in terminal duct lobular units (TDLUs) and resting lymphocytes (having a deep purple–blue rounded nucleus with a small amount of light blue cytoplasm) (*n* = 10) were used as reference cells for comparison.

### Slide scanning

Haematoxylin and eosin (H&E)‐stained slides were digitally scanned using high‐throughput scanners, Leica Aperio AT2 (Leica Biosystems, Buffalo Grove, IL, USA) at 20× or 40× objective magnification (0.5 or 0.25 μm/pixel) or Pannoramic 250 Flash III (3DHistech, Budapest, Hungary) at 40× objective magnification (0.19 μm/pixel). Whole slide images (WSIs) were reviewed on the vendor's respective WSI viewer software, Aperio ImageScope version 12.4.3 from Leica or CaseViewer version 2.4 from 3DHistech, respectively. There were no differences between scanners or images scanned by different magnifications, as described previously.^17^

### Nuclear identification, segmentations and measurements

Two methods were used for nuclear measurements in this study: automated detection method and manual annotation method.

**Figure 2:**
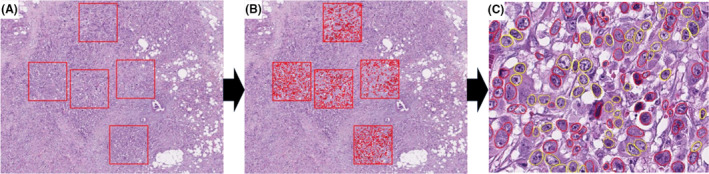
Image analysis using automated methods in this study. **A**, Region of interest annotation in QuPath on whole slide images. **B**, Applying StarDist nuclear detection extension on all annotation areas. **C**, Enlarged view of all detected nuclei (red) and exported nuclei with accurate nuclear detections (yellow). [Color figure can be viewed at wileyonlinelibrary.com]

In the automated detection method for nuclear extraction, QuPath image analysis software^18^ version 0.5.0 was used (Figure 2). A new project was first created, WSIs were imported into the project and the image type was assigned brightfield (H&E) images. Stain normalisation for achieving better colour deconvolution was carried out using the ‘estimate stain vector’ command, which was applied to the whole project. One to five regions of interest (ROI) rectangles, 2000 × 2000 pixels each, were annotated for each slide. Then, StarDist extension^19^ (a deep‐learning‐based method of 2D nucleus detection) was applied for nuclear detection. Detected nuclei were further classified into tumour/immune cells and measurements were exported. Quality check was applied to all nuclear detections, and only accurate detections were exported for analysis. Cell detections were not applied, as indistinct cell membrane was noticed in most of the cases. As a validation for the automated detection method, an initial exhaustive manual nuclear annotation of 30 cases was carried out. We used the same method for extracting and measuring nuclei as in our previous study.^20^ Briefly, ROI images for each breast lesion and normal cells were captured at 120× digital magnification with pixel size 2532 × 1605. These images were analysed using ImageJ version 1.53a (https://imagej.nih.gov/ij/) by outlining all non‐overlapping nuclei with easily detectable boundaries using a digital pen and tablet (Microsoft, Redmond, WA, USA) (Supporting information, Figure S1). After setting the spatial scale of images in ImageJ, measurement results were presented in micrometres (μm).

Nuclear measurements encompassing nuclear size parameters (area, perimeter, maximum Feret's diameter and minimum Feret's diameter) were exported. Calculation of the median values and interquartile range (IQR) for each parameter was used throughout the study to display the nuclear size and nuclear size variability, respectively. The definition of assessed parameters was summarised in Supporting information, Table S1.

### Visual assessment

An additional set of pILC cases (*n* = 90) was visually assessed to characterise cellular and subcellular features as added features to those detected using image analysis. Those features include nuclear size, shape, membrane irregularity, chromatin pattern, nucleolar prominence, cytoplasmic abundance and cytoplasmic eosinophilia.

### Statistical analysis

Statistical analysis was performed using SPSS version 28. The description of continuous variables was performed using median and IQR. Data were checked for normality before statistical analysis using the Kolmogorov–Smirnov test. Based on the results, appropriate statistical methods were chosen: Student's *t*‐test and Mann–Whitney *U*‐test were employed for normally and non‐normally distributed data, respectively, to compare nuclear morphological parameters across different histological types. Receiver operating characteristic (ROC) curves were used to calculate the sensitivity, specificity and area under the curve (AUC), and to determine the cut‐off values for nuclear size parameters derived between classic and pleomorphic subtypes. The cut‐off value with the highest sensitivity and specificity values was considered. Additionally, multivariable logistic regression was employed to assess the independent predictive value of each nuclear morphological parameter. A *P‐*value ≤ 0.05 (two‐tailed) was considered statistically significant.

This work obtained ethics approval by the North West—Greater Manchester Central Research Ethics Committee under the title Nottingham Health Science Biobank (NHSB), reference number 15/NW/0685. Data collected in this study were fully anonymised.

## Results

### Comparison with manual and automated methods

An initial analysis of the study cohort using both visual (eyeballing) and automated (image analysis) methods for a set of cases diagnosed as cLC (both *in situ* and invasive lesions) was carried out. Comparison analysis revealed no statistically significant differences between manual and automated methodologies (Supporting information, Table S2). Subsequently, the entire cohort was analysed utilising the automated image analysis method (QuPath), generating nuclear measurements for 24 404 nuclei. The number of cases and examined nuclei per lesion included in the study are summarised in Supporting information, Table S3.

### Nuclear size in lobular carcinoma *in situ* and invasive lobular carcinoma variants

Table 1 summarises the nuclear size of each LC variant. cLCIS showed a median nuclear area of 32.7 μm^2^ (25.9–40.0) with a narrow IQR (13.8), which is similar to the median nuclear area of cILC with pleomorphism score 1. Meanwhile, pLCIS showed a median nuclear area of 53.0 μm^2^ (41.1–67.9) with larger IQR (26.2), exhibiting intermediate results between ILC with pleomorphism scores 2 and 3.

**Table 1:** Nuclear size (in μm) in lobular carcinoma variants

	Area median (25–75th percentile)	Area IQR	Perimeter median (25–75th percentile)	Perimeter IQR	Min diam median (25–75th percentile)	Min diam IQR	Max diam median (25–75th percentile)	Max diam IQR
LCIS								
Classic variant	32.7 (25.9–40.0)	14.1	21.0 (18.8–23.3)	4.5	5.7 (4.9–6.3)	1.4	7.5 (6.7–8.5)	1.8
Pleomorphic variant	53.0 (41.1–67.9)	26.2	27.1 (24.0–30.4)	6.4	7.0 (6.1–8.1)	2.0	9.9 (8.8–11.2)	2.4
ILC								
Classic, pleomorphism score 1	34.1 (28.0–41.7)	13.7	21.2 (19.3–23.6)	4.3	5.8 (5.2–6.4)	1.2	7.6 (6.8–8.5)	1.7
Classic, pleomorphism score 2	43.0 (34.7–52.2)	17.5	24.0 (21.5–26.6)	5.1	6.4 (5.8–7.2)	1.4	8.6 (7.6–9.7)	2.1
Solid, pleomorphism score 2	40.4 (33.8–48)	14.3	23.1 (21.1–25.1)	4.0	6.5 (5.9–7.1)	1.2	8.1 (7.3–8.9)	1.6
Pleomorphic, pleomorphism score 3	66.6 (50.1–86.4)	36.3	30.0 (26.2–34.2)	8.0	7.9 (6.7–9.2)	2.5	10.9 (9.4–12.5)	3.1

diam, diameter; ILC, invasive lobular carcinoma; IQR, interquartile range; LCIS, lobular carcinoma *in situ*.

In ILC, while pleomorphism score 1 showed exclusively classic growth patterns, a diverse range of architectural patterns, including classic and solid, was observed in cases showing pleomorphism scores 2 and 3. Pleomorphism score 3 is characterised by more pronounced cellular atypia with abundance of cytoplasm, often seen in morphologies such as apocrine and pleomorphic non‐apocrine types. Focal areas displaying histiocytoid and signet‐ring cell morphology seen in some pILC cases typically showed nuclear pleomorphism score 2 rather than score 3. In this study, we focused upon classifying ILC variants based on nuclear pleomorphism with a particular emphasis on capturing the characteristics of the pleomorphic variant, categorising cILC with scores 1 and 2, solid ILC (sILC) with score 2 and pILC with score 3.

In cILC, tumours with pleomorphism score 1 showed a median nuclear area of 34.1 μm^2^ (28.0–41.7), with the narrowest IQR (13.7). In contrast, pILC (pleomorphism score 3) showed greater variability in the nuclear area, with the largest IQR (36.4); the median nuclear area was 66.6 μm^2^ (50.1–86.4). In pleomorphism score 2, both cILC and sILC exhibited similar values. pILC cells showed a median nuclear area of 1.6–2.0 times larger than that of cILC cells, highlighting significant differences in nuclear size between these lobular carcinoma variants.

To differentiate between cILC or sILC and pILC, we established optimal cut‐off values for various nuclear morphological parameters using ROC curves. This was conducted on nuclear size as well as on other variability metrics. Cut‐offs based on the median values of nuclear measurements were as follows:

- Area = 48.2 μm^2^ (AUC = 0.83, sensitivity = 77.6%, specificity = 75%).
- Perimeter = 25.2 μm (AUC = 0.84, sensitivity = 80%, specificity = 76%).
- Minimum diameter = 6.8 μm (AUC = 0.77, sensitivity = 72%, specificity = 70%).
- Maximum diameter = 9.1 μm (AUC = 0.85, sensitivity = 80%, specificity = 76%).

As an indicative of nuclear size variability (pleomorphism), cut‐offs of IQR were shown as follows:

- Area IQR = 19.4 (AUC = 0.92, sensitivity = 92%, specificity = 92%).
- Perimeter IQR = 5.3 (AUC = 0.92, sensitivity = 71%, specificity = 86%).
- Min diameter IQR = 1.5 (AUC = 0.94, sensitivity = 86%, specificity = 89%).
- Max diameter IQR = 2.2 (AUC = 0.93, sensitivity = 71%, specificity = 92%).

Multivariable logistic regression analysis confirmed that each nuclear size parameter independently contributes to the diagnosis of pILC (Table 2). All parameters demonstrated significant predictive value in distinguishing pILC from cILC or sILC (*P* ≤ 0.05), with the maximum diameter emerging as the most influential factor (odds ratio = 5.33, 95% confidence interval = 4.57–6.22, *P* < 0.001).

**Table 2:** Logistic regression multivariable analysis for identifying pILC

Nuclear size parameters	Odds ratio	95% confidence interval	*P*‐value
Area	1.44	1.11–1.87	0.006
Perimeter	1.59	1.21–2.09	<0.001
Min diam	1.54	1.35–1.77	<0.001
Max diam	5.33	4.57–6.22	<0.001

diam, diameter; pILC, pleomorphic invasive lobular carcinoma.

### Nuclear size in invasive lobular carcinoma variants compared to normal cells and score‐matched IBC‐NST

Table 3 shows the relative magnitude of ILC variants in comparison to the nuclear size of normal epithelial cells, lymphocytes and pleomorphism score‐matched IBC‐NST. Cells of cILC with pleomorphism score 1 were 2.0 times larger than resting lymphocytes, 1.5 times normal epithelial cells and 1.1 times the score‐matched IBC‐NST in the median nuclear area. The pleomorphism score 2 cILC cells was 2.5 times that of resting lymphocytes and 1.8 times of normal epithelial cells, and it was 1.1 times of score‐matched IBC‐NST in the median area. In pleomorphism score 2 sILC we observed a similar trend to classic architecture, with the median area 2.3 times that of resting lymphocytes and 1.7 times that of normal epithelial cells, and it was equivalent to the score‐matched IBC‐NST in the median area. The median nuclear area of pILC cells was 3.8 times compared to resting lymphocytes, 2.8 times to normal epithelial cells and 1.3 times to pleomorphism score 3 IBC‐NST.

**Table 3:** Nuclear size (in μm) in invasive lobular carcinoma variants compared to normal cells and score‐matched IBC‐NST

	Relation to lymphocytes (times)				Relation to normal epithelial cells (times)				Relation to score matched IBC‐NST (times)			
	Area	Perimeter	Min diam	Max diam	Area	Perimeter	Min diam	Max diam	Area	Perimeter	Min diam	Max diam
Classic, pleomorphism score 1	2	1.4	1.4	1.4	1.5	1.2	1.2	1.2	1.1 (*P* < 0.001)	1.1 (*P* < 0.001)	1 (*P* < 0.001)	1.1 (*P* < 0.001)
Classic, pleomorphism score 2	2.5	1.6	1.5	1.6	1.8	1.3	1.3	1.3	1 (*P* = 0.02)	1 (*P* = 0.4)	1 (*P* = 0.4)	1 (*P* = 0.01)
Solid, pleomorphism score 2	2.3	1.5	1.5	1.5	1.7	1.3	1.4	1.3	1 (*P* = 0.02)	1 (*P* = 0.004)	1 (*P* = 0.9)	1 (*P* < 0.001)
Pleomorphic, pleomorphism score 3	3.8	2	1.9	2.1	2.8	1.7	1.6	1.7	1.3 (*P* < 0.001)	1.2 (*P* < 0.001)	1.1 (*P* < 0.001)	1.2 (*P* < 0.001)

IBC‐NST, invasive breast cancer of no special type; diam, diameter; ILC, invasive lobular carcinoma.

### In‐depth examination of pleomorphic ILC

Table 4 shows an individual presentation of the median nuclear area of all pILC. Of the 14 examined cases in pILC, three cases exhibited apocrine change. A statistically significant difference in the median nuclear area was found between cases featuring apocrine morphology and those lacking a special cytomorphology, where apocrine morphology was associated with a smaller median nuclear area (*P* < 0.001).

**Table 4:** Individual presentation of all 14 pleomorphic ILC

	Area median (25–75th percentile)	Area IQR	Special cytomorphology	Relation to lymphocytes (times)	Relation to normal epithelial cells (times)	Relation to score‐matched IBC‐NST (times)
Case 1	75.7 (57.4–98.2)	40.7	–	4.4	3.2	1.5
Case 2	47 (29.0–66.1)	37.1	–	2.7	2.0	0.9
Case 3	56.2 (46.6–67.5)	20.9	–	3.2	2.4	1.1
Case 4	76.7 (58.9–94.7)	35.8	–	4.4	3.3	1.5
Case 5	82.5 (60.5–107.9)	45.4	–	4.7	3.5	1.6
Case 6	80.7 (65.0–92.8)	27.8	–	4.6	3.6	1.5
Case 7	76.2 (58.4–96.4)	38	–	4.4	3.3	1.5
Case 8	61.9 (46.7–75.9)	28.9	Apocrine	3.6	2.6	1.2
Case 9	58.7 (48.3–70.3)	22	–	3.4	2.5	1.2
Case 10	62.4 (52.1–75.7)	23.7	Apocrine	3.6	2.7	1.2
Case 11	62.0 (52.9–63.9)	21	Apocrine	3.6	2.6	1.2
Case 12	51.5 (36.7–72.3)	35.6	–	3.0	2.2	1.0
Case 13	50.4 (40.0–61.6)	21.6	–	3.0	2.1	1.0
Case 14	52.5 (30.3–63.1)	32.8	–	3.0	2.2	1.0

IBC‐NST, invasive breast cancer of no special type; IQR, interquartile range.

Notably, the maximum median nuclear area in relation to a lymphocyte was less than 5 (4.7) times. pILC median nuclear area varied from 2.7 to 4.7 median lymphocyte size, 2.0 to 3.6 normal ductal epithelial cells and 0.9 to 1.6 NST score 3 median nuclear area.

### Visual assessment of the additional pleomorphic ILC cohort

In the visual assessment of 90 cases of pILC, various cytomorphological and architectural characteristics were identified. The pleomorphism score 3 was predominant in tumour tissue in 76% of cases, while it was a less predominant component (< 50%) in 24% of cases. Most of them showed a classic growth pattern, invading as separate cells and single files, while 17% included a solid growth pattern. Additionally, 20% of them showed a signet‐ring cell like component.

Nuclear size in pILC mainly comprised a mixture of different‐sized small and large cells, observed in 79% of the cases. An irregular nuclear membrane was predominant in 75% of the cases and a vesicular nuclear pattern was seen in 81%. Prominent nucleoli were observed in 32% of the cases. Cytoplasmic abundance and marked eosinophilia were noted in 42 and 44% of the cases, respectively. However, most cases lacked a distinct nuclear membrane, complicating the accurate assessment of the cytoplasmic and nuclear‐cytoplasmic ratio.

**Figure 3:**
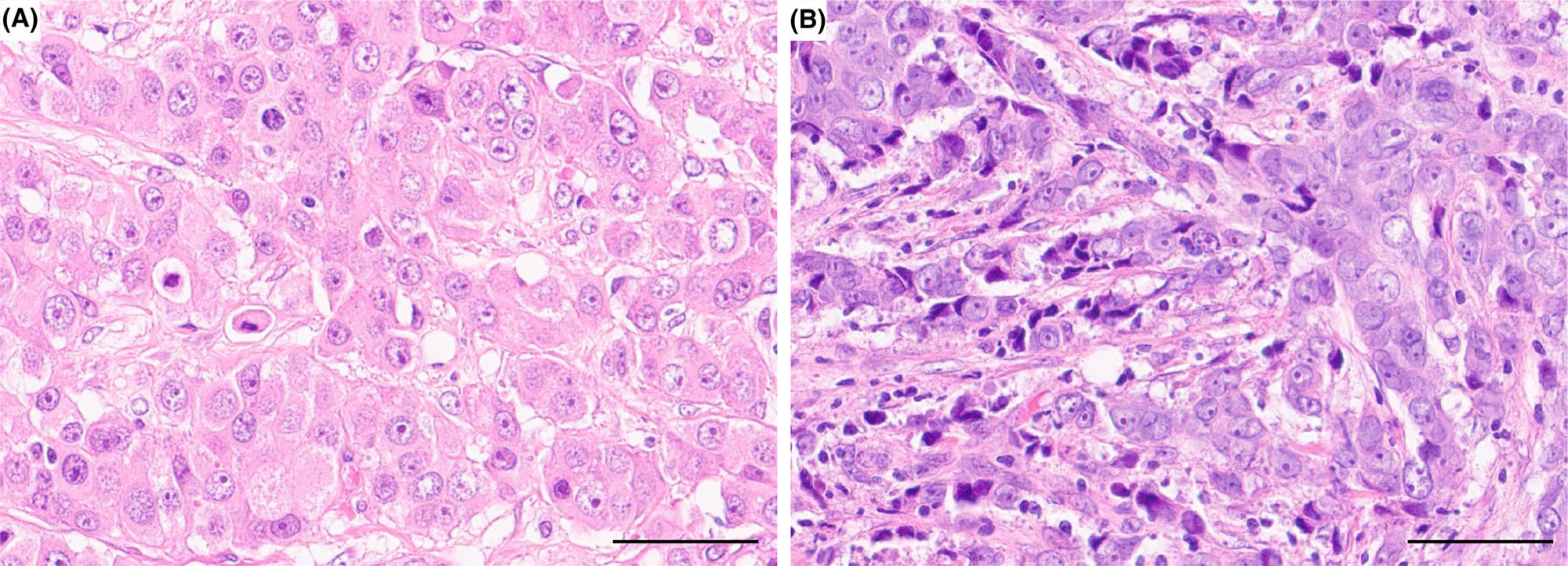
Two subtypes of pleomorphic invasive lobular carcinoma. **A**, Apocrine subtype with pale vesicular nuclei with prominent nucleoli and abundant eosinophilic cytoplasm. **B**, Non‐apocrine subtype with prominent dark stained smudged nuclei. Haematoxylin and eosin, scale bar = 50 μm). [Color figure can be viewed at wileyonlinelibrary.com]

Focusing upon cytomorphology variants, two pILC subtypes were recognised: apocrine and non‐apocrine (also known as ‘lacking special cytomorphology’) (Figure 3). Twenty per cent of cases displayed typical apocrine features and were classified as the apocrine subtype. This subtype significantly featured predominantly large‐sized cells with more or less round‐shaped nuclei, minimal nuclear membrane irregularity, fewer variations in size, pale vesicular nuclei with prominent nucleoli and abundant eosinophilic cytoplasm compared to the non‐apocrine subtype (*P* < 0.001). In contrast, the non‐apocrine subtype showed greater variation in nuclear size and shape, more darkly stained and smudged chromatin patterns and less cytoplasmic abundance. Table 5 summarises the differences between these two subtypes. Distinguishing between the two subtypes required careful scrutiny of cytoplasmic features, such as abundant eosinophilia and vacuolated, foamy cytoplasm, in conjunction with nuclear observations.

**Table 5:** Features for distinguishing two subtypes of pleomorphic invasive lobular carcinoma

Features	Apocrine (20%)	Non‐apocrine (80%)
Nuclear size	Large	Mixed small and large
Nuclear shape	Almost round	Variable
Nuclear membrane irregularity	Minimal	Marked
Variability in nuclear size and shape	Minimal variability	High variability
Chromatin pattern	Pale vesicular and rare smudged nuclei	Vesicular with marked association with dark or smudged nuclei
Prominent nucleoli	More predominant	Predominant
Cytoplasmic abundance	Predominant	Less predominant
Cytoplasmic eosinophilia	Predominant	Less predominant

## Discussion

In this study, we provide data on nuclear features with defined cut‐offs to improve the diagnostic concordance of LC subtype classification and to ensure best patient care in view of the prognostic difference among these subtypes.^21^ Although nuclear size is an important diagnostic criterion our results indicated that other features, such as nuclear pleomorphism (variability) and cytoplasmic characteristics, can further refine subtype classification. Currently, no defined cut‐offs or evidence‐based criteria exist for the nuclear and cytoplasmic characterisation of LC variants, with subtype classification largely based on subjective qualitative features.^5^, ^10^

Nuclear size has proved to be a crucial diagnostic marker in breast cancer, offering a quantifiable measure to aid in diagnosis.^20^, ^22^, ^23^ In particular, pILC's pronounced increase in nuclear size is the key diagnostic feature.^5^, ^10^ We found that pILC exhibits not only markedly larger but also more variable nuclear morphological parameters, including nuclear area, perimeter and maximum/minimum diameter, in addition to nuclear hyperchromasia, prominent nucleoli and cytoplasmic abundance compared to cILC. The median, as a cut‐off for nuclear size, can be considered a practical starting point; however, given the variability within pILC, more refined criteria such as quartiles or percentiles may capture the range of pleomorphism more effectively. To differentiate more objectively between cILC or sILC and pILC, our results suggest cut‐off criteria that incorporate both the median and IQR, which were further validated through multivariable logistic regression analysis. All parameters demonstrated significant predictive value in distinguishing pILC from cILC or sILC, with the maximum diameter emerging as the most influential factor. These findings highlight the robustness of these morphological features in differential diagnosis and propose a more objective approach to diagnosing pILC.

The thorough comparison with normal cells and IBC‐NST also provided a valuable context for interpreting ILC variants. Similar to the previous study by Sneige *et al*.,^24^ we showed the nuclei of pleomorphic type were often more than 3.8 times the median nuclear area of lymphocytes. However, Sneige *et al*.^24^ did not mention which nuclear size parameters were used for comparison. According to our results, the median maximum Feret's diameter of the pleomorphic type nuclei were 2.1 times larger than those of lymphocytes and 3.8 times of lymphocytes nuclei median area. Sneige *et al*.^24^ reported sizes up to six times that of lymphocytes. Nevertheless, our study did not observe cases with such substantial enlargement in the mean area of all 14 pILC cases.

While nuclear size is a vital factor, it is essential to consider additional morphological features to improve diagnostic accuracy. Visual assessment showed that pILC subtype includes apocrine and non‐apocrine. The frequency of cytomorphology subtype observed in our cohort was 80% non‐apocrine and 20% apocrine, consistent with previous findings.^25^ pILC with apocrine features has been reported to be associated with worse prognosis,^25^ emphasising the need to evaluate cytoplasmic characteristics carefully. To distinguish between apocrine and non‐apocrine subtypes, it was crucial to examine cytoplasmic characteristics carefully, including abundant eosinophilia and vacuolated, foamy cytoplasm, in addition to nuclear observations.

Although the 5th edition of the WHO classification of breast tumours demonstrates that pLCIS cells size up to four times that of lymphocytes,^5^ our study did not observe such significant enlargement in the median area. The median nuclear area was 3.0 times that of lymphocytes, ranging from 1.1 to 12.6 times. This suggests that nuclear size alone may not be sufficient for LCIS classification and a multifactorial approach, including nuclear pleomorphism and structural distortions, is necessary for accurate diagnosis.

Our attempt to identify objective nuclear morphological features for distinguishing ILC from pleomorphism score‐matched IBC‐NST suggests that this difference may be minimal and difficult to recognise visually. Focusing upon the nuclear area, pILC demonstrated a mean area 1.3 times larger than score‐matched IBC‐NST, exhibiting a statistically significant difference. Although this difference is subtle, it may be beneficial to explore ways to visualise or quantify this distinction more clearly. One approach could be to enhance image contrast or employ advanced image processing techniques to highlight the differences. Furthermore, combining nuclear area measurements with other morphological parameters such as nuclear shape and chromatin texture may improve the accuracy of distinguishing these entities. There have only been a few previous reports that addressed nuclear morphology of LC,^24^, ^26^ although the usefulness of this parameter remains unclear. The growth pattern in single files or loosely cohesive clusters and loss of E‐cadherin protein expression by IHC are the hallmark diagnostic features of LC and are useful for distinction from ductal lesions.^1^, ^5^, ^10^ However, some ILC/LCIS showed aberrant E‐cadherin membranous expression: incomplete membrane staining, weak complete membrane staining and focal staining patterns, accounting for 15% of ILCs.^27^ In some challenging cases showing aberrant E‐cadherin expression, solid growth and high nuclear‐grade tumours, new parameters are required to determine the diagnosis of LC.

Furthermore, pre‐analytical factors such as tissue fixation, staining quality, and scanning can significantly influence nuclear morphology and subsequent measurements.^28^ Standardisation of these pre‐analytical processes is crucial for ensuring reproducibility and accuracy in nuclear measurements.^29^, ^30^ Another challenge lies in the robustness and validation of image analysis software, as well as the level of training required for pathologists to utilise these tools effectively. Ensuring that pathologists receive adequate training and that the software undergoes rigorous validation will be critical to successfully implementing these techniques in routine clinical practice. Addressing these factors, together with further research, will enhance the application of our findings and lead to more reliable, standardised diagnostics for LC subtypes.

The study has some limitations. First, we could not analyse the entire tumour area, which may lead to sampling bias due to intratumoural heterogeneity. This could affect the representativeness and generalisability of our findings. Future studies should include a more comprehensive analysis of the whole tumour to understand this variability more clearly. Secondly, although the number of pILC cases in the present study is greater than in previous studies of pILC, further validation is necessary to clarify whether the findings from this study improve the diagnosis and definition of pILC. Future research should focus upon refining measurement criteria utilising larger data sets with outcome data, advancing imaging techniques to improve visualisation and accuracy and combining morphological features with molecular data to provide clearer diagnostic insights.

In conclusion, our study highlights the importance of considering nuclear pleomorphism and cytomorphology together with nuclear size in characterising variants of LC. Our findings suggest a need for integrating multiple morphological parameters and further research to elucidate their diagnostic utility in LCs.

## Conflicts of interest

The authors have no conflicts of interest to declare.

## Supporting information


**Figure S1.** Image analysis using manual methods in this study. Nuclear extraction by handwriting. ROI image of outlined tumour nuclei. (HE, Scale bar = 50 μm).

**Table S1.** Description of the studied nuclear morphological parameters.

**Table S2.** Comparison with manual and automated methods for nuclear size measurements.

**Table S3.** Number of the various lobular carcinomas included in this study and the total number of extracted nuclei.

## Data Availability

The authors confirm the data that have been used are available upon reasonable request.
