# Supplementary figures and images for: Nuclear morphological characterisation of lobular carcinoma variants: a morphometric study
Source: Histopathology. 2024 Dec 9;86(5):813–23. doi: 10.1111/his.15390 (PMC11903112; doi:10.1111/his.15390)

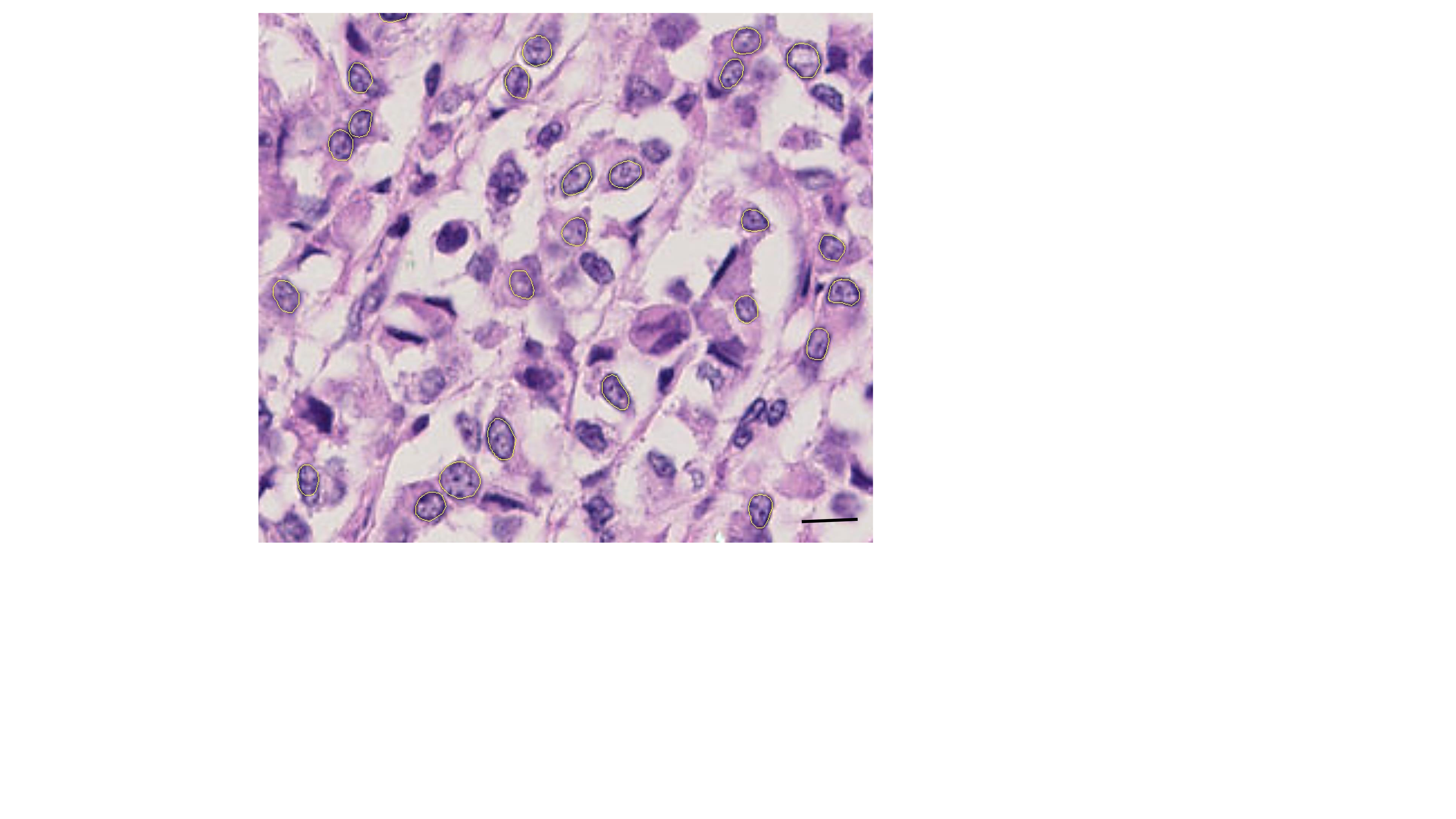

Supplement: Supplementary file 1 — Figure S1. Image analysis using manual methods in this study. Nuclear extraction by handwriting. ROI image of outlined tumour nuclei. (HE, Scale bar = 50 μm). [file HIS-86-813-s002.tif]
