# Supplementary material for: Nuclear morphological characterisation of lobular carcinoma variants: a morphometric study
Source: Histopathology. 2024 Dec 9;86(5):813–23. doi: 10.1111/his.15390 (PMC11903112; doi:10.1111/his.15390)
Supplement: Supplementary file 2 — Table S1. Description of the studied nuclear morphological parameters. [file HIS-86-813-s001.docx]

**Supplementary Table 1.** Description of the studied nuclear morphological parameters

| Parameters | Measurements | Comments |
| --- | --- | --- |
| **Nuclear size** | | |
| Maximum/Minimum Feret's diameter | The longest/shortest distance between any two points along the selection boundary | Also known as maximum/minimum calibre |
| Major/Minor axis lengths | Computed from the fitted ellipse to the object |  |
| Perimeter | Total boundary |  |
| Area | Real area of the object |  |
