# Supplementary material for: Nuclear morphological characterisation of lobular carcinoma variants: a morphometric study
Source: Histopathology. 2024 Dec 9;86(5):813–23. doi: 10.1111/his.15390 (PMC11903112; doi:10.1111/his.15390)
Supplement: Supplementary file 3 — Table S2. Comparison with manual and automated methods for nuclear size measurements. [file HIS-86-813-s003.docx]

**Supplementary Table 2. Comparison with manual and automated methods for nuclear size measurements**

|  | **Manual annotation Method** | | | | | | | **Automated detection Method** | | | | |  |  |
| --- | --- | --- | --- | --- | --- | --- | --- | --- | --- | --- | --- | --- | --- | --- |
| Parameters | Normal cells^*^ | **Classic LCIS** | Relation to normal cells (times) | **Classic ILC, pleomorphism score 1** | Relation to normal cells (times) | **Classic ILC, pleomorphism score 2** | Relation to normal cells (times) | Normal cells^*^ | **Classic LCIS** | Relation to normal cells (times) | **Classic ILC, pleomorphism score 1** | Relation to normal cells (times) | **Classic ILC, pleomorphism score 2** | Relation to normal cells (times) |
|  |  | (total　extracted nuclei 1,485) |  | (total　extracted nuclei 1,408) |  | (total　extracted nuclei 2,362) |  |  | (total　extracted nuclei 1,150) |  | (total　extracted nuclei 1,608) |  | (total　extracted nuclei 2,185) |  |
| Area (μm^2^) | Epithelial cells in TDLUs | 30.48 (26.01–36.79) | 1.3 | 31.25 (26.61–36.75) | 1.4 | 42.69 (34.89–52.90) | 1.9 | Epithelial cells in TDLUs | 32.7　(25.9-40.0) | 1.4 | 34.1　(28.0-41.7) | 1.5 | 43.0　(34.7-52.2) | 1.8 |
|  | 22.99 (19.88–26.46) |  |  |  |  |  |  | 23.4　(19.2-28.4) |  |  |  |  |  |  |
|  | Resting lymphocytes |  | 2 |  | 2.0 |  | 2.8 | Resting lymphocytes |  | 1.9 |  | 2.0 |  | 2.5 |
|  | 15.47 (13.72–17.55) |  |  |  |  |  |  | 17.4　(15.7-18.8) |  |  |  |  |  |  |
| Maximum Feret’s diameter (μm) | Epithelial cells in TDLUs | 7.29 (6.64–8.03) | 1.1 | 7.36 (6.74–8.00) | 1.1 | 8.63 (7.77–9.54) | 1.3 | Epithelial cells in TDLUs | 7.5　(6.7-8.5) | 1.2 | 7.6　(6.8-8.5) | 1.2 | 8.6　(7.6-9.7) | 1.3 |
|  | 6.66 (6.11–7.25) |  |  |  |  |  |  | 6.4　(5.8-7.1) |  |  |  |  |  |  |
|  | Resting lymphocytes |  | 1.4 |  | 1.4 |  | 1.7 | Resting lymphocytes |  | 1.4 |  | 1.4 |  | 1.6 |
|  | 5.09 (4.75–5.43) |  |  |  |  |  |  | 5.3　(5.1-5.6) |  |  |  |  |  |  |

* Median (25th-75th percentile)
