# Supplementary material for: Nuclear morphological characterisation of lobular carcinoma variants: a morphometric study
Source: Histopathology. 2024 Dec 9;86(5):813–23. doi: 10.1111/his.15390 (PMC11903112; doi:10.1111/his.15390)
Supplement: Supplementary file 4 — Table S3. Number of the various lobular carcinomas included in this study and the total number of extracted nuclei. [file HIS-86-813-s004.docx]

**Supplementary Table 3**. Number of the various lobular carcinomas included in this study and the total number of extracted nuclei.

| Diagnosis | Total number of cases | Total number of scored nuclei |
| --- | --- | --- |
| **Lobular carcinoma in situ** | | |
| Classic variants | 4 | 1,150 |
| Pleomorphic variants | 4 | 1,128 |
| **Invasive lobular carcinoma** | | |
| Classic, pleomorphism score 1 | 10 | 1,608 |
| Classic, pleomorphism score 2 | 16 | 2,185 |
| Solid, pleomorphism score 2 | 11 | 3,373 |
| Pleomorphic, pleomorphism score 3 | 14 | 4,233 |
| **IBC-NST** | | |
| Pleomorphism score 1 | 4 | 1,450 |
| Pleomorphism score 2 | 15 | 2,037 |
| Pleomorphism score 3 | 14 | 2,579 |
| **Normal cells** |  |  |
| Lymphocytes | 5 | 1,554 |
| Normal ductal cells | 5 | 3,107 |
